# The Role of Caregiver Mastery in Anxiety Symptoms in People Living With Dementia

**DOI:** 10.1093/geroni/igab046.2094

**Published:** 2021-12-17

**Authors:** Yeji Hwang, Nancy Hodgson

**Affiliations:** 1 University of Pennsylvania, School of Nursing, Philadelphia, Pennsylvania, United States; 2 University of Pennsylvania, School of Nursing, philadelphia, Pennsylvania, United States

## Abstract

Anxiety symptoms in people living with dementia (PLWD) are the most distressing symptoms for caregivers. While caregiving is bidirectional relationship, little is known how caregivers can influence anxiety in PLWD. The purpose of this study was to examine the relationship between caregiver mastery and anxiety symptoms in PLWD. Secondary data analysis was conducted using baseline data from Healthy Patterns Study. The conceptual model of Factors Associated with Behavioral and Psychological Symptoms of Dementia guided this study. Among the 169 study PLWD, 23.1% (n=39) reported having anxiety symptoms. In a multivariate logistic regression, adjusting for age, dementia stage, sleep, and depression, better caregiver mastery was significantly related to lower odds of having anxiety in PLWD (OR=0.87, p=0.046). These results suggest that interventions aimed at improving caregiver mastery may improve anxiety symptoms in PLWD.

